# Target Discovery for Host-Directed Antiviral Therapies: Application of Proteomics Approaches

**DOI:** 10.1128/mSystems.00388-21

**Published:** 2021-09-14

**Authors:** Merve Cakir, Kirsten Obernier, Antoine Forget, Nevan J. Krogan

**Affiliations:** a Quantitative Biosciences Institute (QBI), University of California, San Francisco, California, USA; b Quantitative Biosciences Institute (QBI) COVID-19 Research Group (QCRG), San Francisco, California, USA; c Department of Cellular and Molecular Pharmacology, University of California, San Francisco, California, USA; d Gladstone Institute of Data Science and Biotechnology, J. David Gladstone Institutes, San Francisco, California, USA; Princeton University

**Keywords:** host-directed therapies, systems biology, drug repurposing, host-pathogen interactions, proteomics

## Abstract

Current epidemics, such as AIDS or flu, and the emergence of new threatening pathogens, such as the one causing the current coronavirus disease 2019 (COVID-19) pandemic, represent major global health challenges. While vaccination is an important part of the arsenal to counter the spread of viral diseases, it presents limitations and needs to be complemented by efficient therapeutic solutions. Intricate knowledge of host-pathogen interactions is a powerful tool to identify host-dependent vulnerabilities that can be exploited to dampen viral replication. Such host-directed antiviral therapies are promising and are less prone to the development of drug-resistant viral strains. Here, we first describe proteomics-based strategies that allow the rapid characterization of host-pathogen interactions. We then discuss how such data can be exploited to help prioritize compounds with potential host-directed antiviral activity that can be tested in preclinical models.

## INTRODUCTION

Viral diseases represent a major cause of mortality across the world and can devastate our global health systems (1). The current coronavirus disease 2019 (COVID-19) pandemic illustrates the need to develop innovative tools that allow the rapid identification of potential therapies when new viral epidemics occur. Approaches exploiting mRNA have allowed the development of effective vaccines against COVID-19 at record speed (2–6). While vaccines are often the most efficient approach to control viral infections in the long term, it needs time to be administered to the general population, does not provide 100% antiviral efficiency, and cannot be administered to every patient (7). Therefore, it is critical to develop antiviral therapies as a measure to treat patients suffering from viral diseases when prevention fails. However, drug development is often a process too slow to be readily deployable to an emerging outbreak of disease. It is therefore key to establish new methods that facilitate the rapid identification of antiviral compounds to treat viral infections in vulnerable patients (8).

To date, most FDA-approved antiviral drugs target viral proteins involved in its replication cycle (9). However, a major challenge of these antiviral compounds is that they facilitate the common emergence of drug-resistant viral strains (10, 11) and are effective only against particular infections, hampering the possibility of pan-viral efficacies and repurposing against emerging new diseases. Viruses rely on the host cellular machinery to ensure their replication. Host-directed therapies (HDTs) take advantage of this dependency and attempt to disrupt the virus replication cycle by inhibiting essential host factors (12–17). Host genes that viruses rely on for survival have a low propensity to mutate within the treatment time frame, and adaptation of the virus to compensatory host factors likely occurs only under long-term selection pressure of a host-directed antiviral. Moreover, different viruses may share dependencies of specific host proteins or functions. Therefore, targeting the host proteins required for viral replication is a viable and innovative strategy that can avoid resistance and lead to potentially broad-spectrum therapeutics as families of viruses often exploit common cellular pathways and processes. However, HDTs require in-depth knowledge of virus-host interactions and their biological significance to virus replication. New approaches are aimed at identifying these cellular host factors, and proteomics approaches provide a powerful tool to elucidate the direct physical interactions between host and pathogen as well as perturbations to the host cells proteome caused by viral infection. Using functional genomics, the anti- or proviral function as well as the endogenous function of the identified host factors can be assessed. For example, the role of host factors on viral replication can be studied by knocking out individual host factors using CRISPR followed by viral infection. Only host factors that are identified as host dependency factors without altering critical endogenous molecular functions are suitable for pharmacological inhibition for HDT. Examples of successful HDT include the use of C-C chemokine receptor type 5 (CCR5) antagonists for the human immunodeficiency virus (HIV), cyclosporine for influenza A virus (IAV) and anti-claudin-1 and antioccludin monoclonal antibodies for hepatitis C virus (HCV). CCR5 antagonists inhibit HIV cell entry by blocking the interaction of the HIV type 1 (HIV-1) gp120 envelope glycoprotein with one of its host coreceptors, CCR5 (18, 19). Cyclosporine targets the interaction between influenza virus protein M1 and the host factor cyclophilin A, increasing the ability of cyclophilin A to inhibit M1 (20). It has also been shown that cyclosporine can inhibit nuclear export of viral RNA (21). HCV can bind to the tight junction protein claudin-1 and occludin to enter cells, and monoclonal antibodies against those two proteins have been shown to decrease HCV infection (22, 23). However, despite these examples, great effort has not been placed on therapeutically targeting the host in an effort to combat infectious disease.

**FIG 1:**
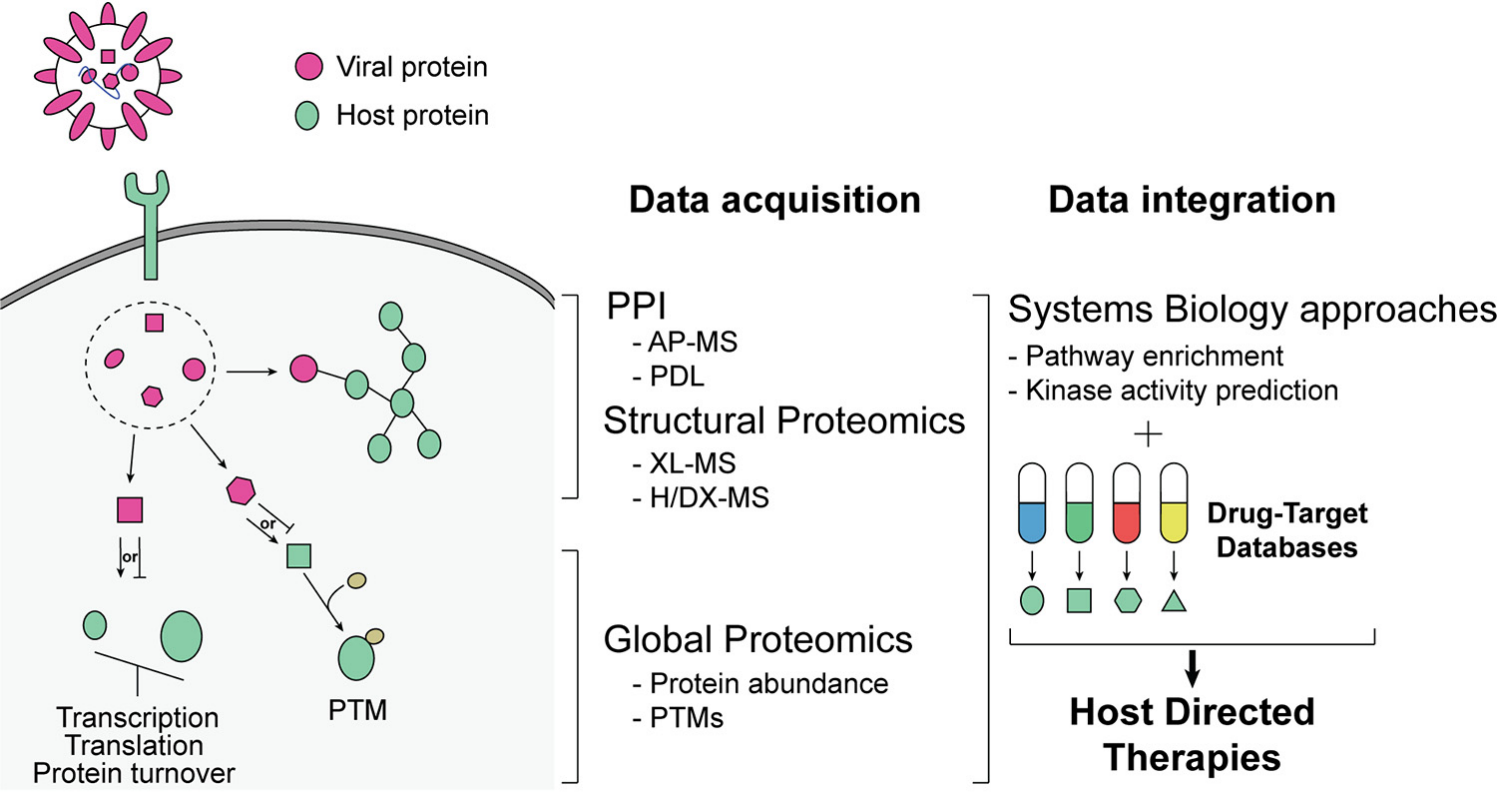
Schematic representation of antiviral host-directed therapy discovery using proteomics and systems biology approaches. AP-MS, affinity purification-mass spectrometry; PDL, proximity-dependent labeling; XL-MS, cross-linking mass spectrometry; H/DX-MS, hydrogen/deuterium exchange mass spectrometry; PTM, posttranslational modification.

Recently, massive efforts to identify therapeutic strategies to manage severe acute respiratory syndrome coronavirus 2 (SARS-CoV-2), the causative agent of COVID-19, have been undertaken. Understanding how SARS-CoV-2 interacts with the host cellular machinery and the identification of host dependency factors have led to the identification of promising drugs and investigational new drugs (INDs) that could be repurposed for HDT (24, 25). In this minireview, we will discuss how proteomics and systems biology approaches can streamline the discovery of antiviral host-directed therapies (Fig. 1). We will first give an overview of the most commonly used proteomics approaches to (i) map viral protein interactions with host proteins, (ii) map the global effects of viral infection on cellular signaling, and (iii) determine structure function. We will then describe how such proteomic data can be used to direct drug repurposing or new drug discovery.

## PROTEOMICS APPROACHES TO IDENTIFY HOST FACTORS

The identification of putative key host factors is crucial to drive the development of innovative HDTs. Proteomics approaches present many advantages to do so, as they allow the unbiased mapping of protein-protein interactions (PPIs) and signaling perturbations due to infection, and ultimately enable structure-function studies. Coupled with other systems biology approaches, proteomic data are particularly suited to guide the discovery of innovative antiviral therapeutic strategies using HDTs (26). This section briefly gives an overview of mass spectrometry (MS) methods used to study virus-host interactions (reviewed extensively in references 27 and 28).

### Proteomics approaches for protein-protein interaction mapping.

Affinity purification-mass spectrometry (AP-MS) and proximity-dependent labeling (PDL) permit the extensive mapping of PPI networks and can uncover host machinery that viral proteins associate with and potentially hijack. Such approaches have successfully been used to map Zika virus, herpesvirus, HIV-1, and SARS-CoV-2 virus-host PPIs and give critical insights into identifying important host factors for those viruses (29–40).

AP-MS is a widely used method to characterize the interactors of epitope-tagged bait proteins (41). Tagged viral proteins are expressed in host cells and purified with their bound interactors that are subsequently identified by MS. AP-MS allows the rapid, quantitative, and unbiased identification of multiple host interactors of a viral protein in a single experiment. While AP-MS presents some limitations, including the potential loss of weak interactions or the difficulty to recover membrane proteins, its ease of use and scalability permit it to generate extensive mapping of PPIs in a very short time. This is exemplified by the timely description of the SARS-CoV-2 interaction network only a few months after the pandemic outbreak (29), an immense undertaking that has been possible due to a massive collaborative effort (42). When designing AP-MS experiments, it is also important to consider that N- or C-terminal tagging can have an effect on protein function, requiring testing the functional status of tagged proteins. Finally, AP-MS also does not discriminate between direct and indirect interactions. However, this can be addressed by coupling AP-MS with cross-linking reagents, which allows the identification of direct interactions (see “Structure MS” section below) (43–45).

PDL uses enzymes fused to the protein of interest to biotinylate interactors in close proximity (46, 47). Two classes of enzymes are mainly used: promiscuous biotin protein ligases (BirA/BioID/TurboID) (48–50) or engineered ascorbic acid peroxidases (APEX) (51–53). In both cases, upon addition of their substrate, proteins in a 10- to 20-nm range are biotinylated and can be subsequently purified using streptavidin resin followed by identification using MS. The main advantages of the PDL approach, compared to AP-MS, resides in its ability to detect transient or weak interactions as well as membrane-bound partners. Moreover, due to the covalent labeling of interacting proteins by biotin, lysis and purification can be performed under stringent conditions to reduce background. PDL can also provide information on the subcellular localization of the identified PPI by the proximal labeling of organelle-specific proteins or specific use of spatial references (54).

### Global proteomics approaches.

Viruses trigger global changes in the molecular landscape of infected cells in order to ensure their replication and evasion from the cell’s innate immunity. Measurements of cell signaling rewiring, regulation of protein levels, and changes to transcription upon viral infection provide a holistic understanding of the mechanisms at play during infection.

High-throughput characterization of posttranslational modifications (PTMs) especially offer valuable insight into the biology of viral infections, as PTMs have critical roles in many different aspects of infection that have both proviral (inhibiting interferon response or promoting viral replication and assembly) and antiviral consequences (degrading viral proteins through ubiquitination or inactivating them through changes in PTMs) (55, 56). Various studies also revealed how the dynamic interplay between different PTMs (such as phosphorylation, ubiquitination, and SUMOylation) regulates processes like pathogen-sensing pathways and innate immune signaling, underscoring the importance of characterizing the dynamics of these modifications upon infection (57–60). In particular, phosphoproteomic profiling of infected cells allows identification of changes in kinase activity over the time course of infection (61–63). Kinases represent attractive drug targets, as many kinase-regulating drugs and compounds have been developed for the treatment of various diseases, and numerous studies have shown that host kinases can regulate various steps of the virus replication cycle (16). Recent improvements in phosphopeptide enrichment using ion metal affinity or ion-exchange chromatography coupled to MS now allows for the routine identification of tens of thousands of phosphorylated peptides in a single experiment (64–66). Moreover, computational methods to quantify and localize the phosphorylation sites have also greatly improved, leading to the possibility to infer many kinases’ activity using growing kinase-substrate relationship databases (67–69). Other PTMs such as ubiquitination or acetylation can be assessed using MS (70–73), and an integrative analysis of multiple PTMs with protein abundance can provide insight into the cross talk between different signaling events (74). Changes in PTM abundance upon infection can lead to the identification of pathways or enzymes controlling such PTMs and targetable using HDTs.

Global protein abundance also provides valuable insights as host proteins can be up- or downregulated after virus infection and show deregulation of pathways that could be therapeutically actionable. Classic global proteomics approaches using mass spectrometry have successfully been used to characterize changes in protein levels following infection (74–76). Recent studies combined global proteomics approaches and thermal proteome profiling (TPP) to assess changes in protein levels and activity during SARS-CoV-2 and cytomegalovirus infection (77, 78). Thermal shift assay approaches, such as cellular thermal shift assays (CESTA), could also be used to understand antiviral compound’s molecular consequences and targets in relevant cell models (79).

Finally, viruses induce reorganization of subcellular structure and organelles of infected cells to promote replication. Such spatial changes in protein levels and organization can be probed using the methodologies discussed here and reviewed in detail by Jean Beltran et al. (80). Briefly, organelle fractionation followed by MS (81, 82) or proximity-based biotinylation (83–87) have been used in numerous studies to assess subcellular compartment content and are particularly suited to study how viruses reshape cell proteome landscape.

### Structure MS.

Cross-linking mass spectrometry (XL-MS) uses chemical cross-linkers to covalently bridge reactive amino acid residues in close proximity (43, 88, 89). Pairs of linked peptides can then be identified by MS. This technique can therefore provide not only PPI information but structural insights on intra- and intermolecular interaction surfaces as well. Combined with dedicated structural approaches such as cryo-electron microscopy (cryo-EM) and structure predictions through deep learning systems such as AlphaFold (Deepmind), XL-MS facilitates and validates structure determination of challenging protein complexes (90, 91). Cross-linking can also be performed in combination with affinity purification of protein baits in order to determine direct interactions between copurifying proteins and improve AP-MS resolution (92).

Hydrogen/deuterium exchange mass spectrometry (H/DX-MS) allows the study of protein conformation for individual proteins or protein complexes. H/DX-MS measures changes in mass associated with the exchange between deuterium isotopes and hydrogens of the protein backbone amides (93, 94). The rate of the exchange is dependent on the conformational state of the protein and surface accessibility. Therefore, H/DX-MS is useful to probe folding dynamics, allosteric changes, protein conformation, and binding sites (95–99). Applications of H/DX-MS include the characterization of protein structure changes in response to PTMs or characterization of the binding of small molecules to proteins.

## DRUG REPURPOSING STRATEGIES FOR DESIGNING ANTIVIRAL THERAPIES

Drug repurposing can offer an expedited timeline to bring host-directed antiviral therapies into clinical settings in a cost-effective and timely manner in comparison to traditional drug discovery, as designing a new compound, characterizing its efficacy, and demonstrating its safety can be a slow and resource-intensive process with a higher risk of failure. Successful applications of repurposing have occasionally been driven by observations of unexpected consequences of drugs, such as the discovery of minoxidil’s effects on hair growth when it was being tested for hypertension and the approval of sildenafil for erectile dysfunction treatment even though it was originally in trial for angina treatment (100, 101). Following advances in various omics technologies and development of computational methodologies, data-driven approaches are also increasingly being used to gain valuable insight into identifying the most suitable candidates for repurposing.

The proteomics approaches described above are suited for repurposing studies, as they enable the identification of host factors and pathways that are hijacked and rewired during infection. Combined with functional genetics to identify host dependency factors, the data obtained provide a rich list of potential targets for therapeutic interventions. Once targets are identified, several databases can be used to determine their druggability and identify existing chemical matter, ranging from preclinical compounds to INDs and FDA-approved drugs. These databases are also used to gain extensive information on relevant drugs, as they catalog their protein targets, structures, chemical properties, or clinical profiles (102). DrugBank is a frequently used database across many studies that make use of drug-target interaction networks and provides detailed information on various properties of more than 14,000 drugs, including their targets, pharmacodynamics, mechanism of action, and toxicity (103). Other valuable resources include DGIdb (the Drug-Gene Interaction Database), ChEMBL, and PharmGKB (the Pharmacogenomics Knowledgebase). DGIdb characterizes the druggable genome by curating information from various drug- and gene-related databases for more than 10,000 drugs and 40,000 genes and annotates their associated drug-target relationships (104). ChEMBL is based on the curation of more than 80,000 publications and offers a broader scope of nearly 2.1 million compounds by providing genomic and chemical data on bioactive drug-like small molecules (105). For studies with a pharmacogenomics focus, PharmGKB is a relevant resource as it curates knowledge on gene-drug associations and effects of genetic variation on drug response (106). Selection of a particular database is typically tailored to specific aims of the approach, and frequently multiple resources are integrated with each other to increase the coverage of drug-gene interaction networks built to connect host factors to relevant drugs.

Several studies have applied proteomics technologies to reveal virus-host interactions and searched for direct interactions between these host factors and drugs, with the aim to identify compounds or drugs that inhibit processes the virus relies on. Studies with this strategy include the work by Dapat et al. on respiratory syncytial virus, where the authors created its interaction network by integrating host factors identified across nine proteomics and seven transcriptomics studies (107). DrugBank was then used to query drugs targeting this network, revealing 177 FDA-approved drugs targeting 78 host proteins belonging to various categories such as anti-infective and anti-cancer agents. An AP-MS study by Gordon et al. identified 332 human proteins interacting with 26 SARS-CoV-2 proteins, revealing various complexes and processes hijacked by the virus, many of which are druggable targets (29). Chemoinformatics approaches and literature search by experts highlighted 69 compounds that target 62 proteins found in the PPI network. Translation inhibitors and molecules that target sigma-1 and sigma-2 receptors emerged as effective antiviral candidates *in vitro*. The translation inhibitor plitidepsin was subsequently characterized as a potent antiviral *in vitro* and *in vivo* with ∼30-fold-higher efficacy than remdesivir (108). Watanabe et al. generated an influenza-human protein interaction network via mass spectrometry and targeted the host factors with small interfering RNAs (siRNAs) to identify those involved in viral replication (109). Querying various drug databases for chemicals targeting these host factors led to the identification of 61 drugs, where further experimental studies highlighted GBF1 inhibitor golgicide A and JAK1 inhibitor ruxolitinib as viable candidates for anti-influenza therapy. Phosphoproteomics approaches (110) have also been successfully used to identify druggable host factors, especially those targeted by kinase inhibitors, which were shown to have antiviral effects against various viruses (111). Dynamics of kinase activity can be inferred computationally following characterization of sites phosphorylated upon infection, and kinases whose activity can explain virus-associated changes in phosphosites are then linked to drugs modulating their activity. This strategy has been applied to various viruses, which led to the nomination of GRK2 inhibitors for influenza (112), JNK1 inhibitors for Japanese encephalitis virus (113), and various inhibitors targeting casein kinase II, p38/MAPK (mitogen-activated protein kinase) signaling, and growth factor receptor signaling for SARS-CoV-2 (61, 114) treatment.

Network-based approaches have also been crucial for nominating drug repurposing candidates by integrating virus-host protein interaction, human protein-protein interaction, and drug-target interaction networks. One key idea behind these approaches is to consider interactions between host factors within a human PPI network and the downstream effects that dysregulation of a protein could cause based on these relationships. These integrative approaches can reveal indirect effects of proteins hijacked by viruses, and searching for drugs that can modulate these downstream effects can present additional druggable opportunities. Network-based studies have especially been valuable for SARS-CoV-2 research, offering resources to identify putative antiviral therapy options in a timely manner. For instance, the study by Zhou et al. generated a coronavirus-host interaction network by integrating various experimental resources that characterized interactions of six different viruses, including SARS-CoV-1 and Middle East respiratory syndrome (MERS)-CoV, to assemble the set of human proteins associated with these viruses (115). This study also generated a drug-target interaction network obtained from various drug databases exemplified above. To identify drugs with the potential to target SARS-CoV-2, Zhou et al. (115) used a “network proximity measure,” which computes distances between the host factors and proteins targeted by a given drug based on the human PPI network’s connectivity and prioritizes drugs whose targets are in close proximity to the host factors.

In another network-based study, Gysi et al. (116) implemented 12 different pipelines that rely on network proximity, network diffusion, and artificial intelligence principles, where they searched for drugs that can target the network of SARS-CoV-2 interactors identified by Gordon et al. (29). Proximity-based approaches focused on the distance between SARS-CoV-2 interactors and targets of drugs to rank drugs based on proximity scores, whereas diffusion methods ranked them based on the network similarity of drug targets and SARS-CoV-2 targets. Graph neural networks were implemented for artificial intelligence-based approaches. After characterizing the results of each individual method, the authors used a multimodal approach to combine different pipelines’ rankings together to generate a consensus list of predictions, taking advantage of the strengths of each individual pipeline. Sadegh et al.’s work offers researchers the opportunity to explore a virus-host interaction network from a drug target identification perspective by developing an online tool called CoVex (Coronavirus Explorer) that provides visualization of virus-host networks, drugs, and their targets (117, 118). They also implemented various network-based algorithms that can build bridges between host factors through neighboring, related proteins and connect these proteins to drugs targeting them, such as multi-Steiner tree, closeness centrality, and degree centrality. Currently, this resource includes PPI networks for SARS-CoV-1 (119, 120) and SARS-CoV-2 (29) with plans to add networks for additional viruses, and it allows the researchers to run custom queries that can start from viral or host proteins of interest to identify a set of drugs that can target them directly or indirectly generating actionable hypotheses on drug repositioning.

Studies highlighted here focused on drug repurposing strategies that have been applied to virus-associated data sets to underscore the utility of these algorithms in identifying antiviral therapies. For additional information on alternative repurposing approaches with a broader scope, we refer readers to several reviews that discuss properties of different algorithms and how various omics technologies and data sources are used for network-based drug repurposing (121–124). Application of these currently available drug repurposing algorithms or development of new approaches specifically tailored for viral proteomics data sets have the potential to identify viable targets for designing effective host-directed antiviral therapies both for viruses that currently lack effective treatments and emergent pathogens.

## OPPORTUNITIES FOR PAN-VIRAL THERAPY DESIGN

Drug repurposing studies we discussed up to now have mostly focused on a single virus and characterizing its set of druggable targets. However, when we compare characteristics of different viruses, commonalities between proteins and biological processes that they interact with start to emerge, raising the possibility of identifying druggable host factors shared across multiple viruses (125). These comparisons could focus on a single family and characterize a set of related viruses in detail while taking their sequence homology and evolutionary differences into account. One such study comparing PPI networks of three coronaviruses revealed the set of interactions that are conserved across the three and trends that are shared between more closely related SARS-CoV-1 and SARS-CoV-2 but not identified in MERS-CoV (126). Drugs targeting proteins shared across viruses were also discussed to exemplify how these networks can be used to discover drugs that can be effective against multiple pathogens. Examples of other comparative analyses include ones that go beyond a single family, such as the study by Pichlmair et al., where they characterized human proteins interacting with 70 viral proteins from 30 different viruses using AP-MS (127) and the study by Bösl et al., which built an interaction network of 17 viruses by curating AP-MS and yeast two-hybrid studies (128). Focusing on a broader range of viruses revealed shared biological processes across distinct viruses that illustrate pathways crucial for viral infection and enabled comparisons between different groups of viruses, such as minus-strand single-stranded RNA [ssRNA(−)], plus-strand ssRNA [ssRNA(+)], double-stranded RNA (dsRNA), and double-stranded DNA (dsDNA) viruses. Additionally, Bösl et al. (128) queried DGIdb to identify drugs targeting proteins within this host-virus interaction network, highlighting putative pan-viral therapy options. Cataloging processes that are unique to individual viruses or shared across viruses will generate a valuable resource as we move forward with the design of host-directed antiviral therapies. It will help prioritize drug repurposing opportunities with the potential to be effective broad-acting antivirals and match a specific set of drugs to the most relevant group of viruses based on the intersection between their targets and shared host factors.

## CONCLUSION

Proteomics and systems biology approaches are strikingly suited to provide a rapid and useful functional landscape of host-virus interactions. While requiring cross discipline expertise, the current available pipelines using these methods can identify important host factors involved in viral replication. Such insights are critical to develop efficient host-targeted antiviral therapies by allowing us to quickly nominate targetable host functions using repurposed drugs or by developing new compounds able to inhibit specific host-virus protein complex formation or activity. Importantly, as the description of different virus interactions with their host partner grows, it becomes clear that commonalities could be identified and exploited to target essential host factors shared by several viruses.
